# Expanding the Methodological Repertoire in Institutional Ethnography: A Design Sociology Approach to Mapping and Visualizing Invisible Work

**DOI:** 10.1111/cars.70020

**Published:** 2025-12-02

**Authors:** Anna Isaksson

**Affiliations:** ^1^ School of Health and Welfare Halmstad University Halmstad Sweden

**Keywords:** design sociology, Institutional ethnography, invisible work, mapping, visualizing

## Abstract

This article presents a novel methodological approach in which institutional ethnography borrows from design sociology. Although mapping is a core component of institutional ethnography, previous research highlights opportunities to further develop mapping techniques as both an analytical tool and a means of presenting research findings. Design sociology is an emerging interdisciplinary field that merges sociological inquiry with design methodologies, offering creative tools for analyzing and visualizing complex social phenomena. The article builds on a project conducted in the elderly care sector, illustrating how design‐based approaches can bring caregivers’ everyday experiences and invisible work to the forefront while revealing systemic challenges. Utilizing design sociology, the study broadens the methodological repertoire of institutional ethnography by introducing new strategies for analysis and communicating research findings. This interdisciplinary framework offers opportunities for mapping and visualizing how people's experiences and activities are structured by larger institutions and structures.

## Résumé

Cet article présente une nouvelle approche méthodologique dans laquelle l'ethnographie institutionnelle s'inspire de la sociologie du design. Bien que la cartographie soit un élément central de l'ethnographie institutionnelle, des recherches antérieures soulignent les possibilités de développer davantage les techniques de cartographie en tant qu'outil analytique et moyen de présenter les résultats de la recherche. La sociologie du design est un domaine interdisciplinaire émergent qui fusionne la recherche sociologique et les méthodologies de conception, offrant des outils créatifs pour analyser et visualiser des phénomènes sociaux complexes. L'article s'appuie sur un projet mené dans le secteur des soins aux personnes âgées, illustrant comment les approches basées sur le design peuvent mettre en avant les expériences quotidiennes et le travail invisible des soignants tout en révélant les défis systémiques. En utilisant la sociologie du design, l'étude élargit le répertoire méthodologique de l'ethnographie institutionnelle en introduisant de nouvelles stratégies d'analyse et de communication des résultats de recherche. Ce cadre interdisciplinaire offre des possibilités de cartographier et de visualiser la manière dont les expériences et les activités des personnes sont structurées par des institutions et des structures plus larges.

## 1 Introduction

Institutional ethnography (IE) focuses on the everyday experiences of individuals as starting points for inquiry. By centering on the actualities of lived experience, IE offers an approach for uncovering the social organization of everyday life. IE seeks to discover how individual actions are coordinated within broader institutional processes, extending beyond what is observable or directly articulated by participants. It emphasizes the translocal relations, the systems and structures that shape and organize daily activities (Smith 2005).

Ontologically, IE rejects abstract theoretical explanations in favor of individuals’ actual practices and activities. Epistemologically, it acknowledges reflexivity and rejects objective accounts of reality. IE seeks to understand the complexity of social relations, how things work, and how they are put together in everyday life (Smith 1987, 1999, 1990; Kearney et al. 2018).

Institutional ethnographers utilize a range of methodological approaches, including interviews, participant observation, and text analysis, to investigate the complex coordination of social relations. Central to this approach is the pivotal role of texts and discourses, which are understood as organizing mechanisms that shape social relations and mediate individuals’ interactions with people, practices, and institutions (Smith 2001, 2005).

A cornerstone of IE is the practice of *mapping*, a process of identifying and visually representing the ways institutional texts and practices structure individuals’ experiences (Smith and Griffith 2022; Turner 2006; Campbell and Gregor 2004). Mapping reveals connections among diverse and distinct local sites of experience. A map can be revised and corrected (Smith 1999; DeVault 2023). It is iterative and begins with the localized, everyday experiences of individuals and expands outward by linking them to broader ruling relations, such as institutional discourses and practices that influence and organize those experiences (Smith 1990; Smith and Griffith 2022).

Another cornerstone of IE is the interest in and recognition of *invisible work*. This term refers to the often unacknowledged and undervalued labor performed, particularly by women, in the domestic sphere and caregiving roles. This labor sustains societal functions yet remains largely unrecognized in policy documents, economic and organizational terms (Smith 1987; Ingraham and Dacher 2014 referenced in Devault 2014). A central area of inquiry for IE is, therefore, how bureaucratic systems and societal norms perpetuate the invisibility of this type of work (Smith 1987, 1990, 2005).

In this article, special attention is given to how a design sociology approach can be applied in an IE‐oriented study to map and visualize invisible work. Generally, design is seen as the process of developing targeted solutions intended for real‐world application. However, in this article, the term design refers specifically to the act of designing as an integral part of the research process. Rather than serving a purely practical function, design‐based activities are recognized for their role in shaping and informing the creation of new knowledge. This conception of design originates from the field of Research through Design (RtD). In RtD, design activities take various forms that contribute to knowledge generation. These include creating stimulus materials, which are simple design outputs used to support other researchers' work. Concept development and ideation involve navigating between theory and practice, balancing constraints and opportunities as designers shape meaningful prototypes. Finally, prototyping is a central design activity that entails developing artifacts which may resemble products but are intended to provoke reflection, dialogue, or novel interactions (Stappers and Giaccardi 2014).

Design sociology is an emerging interdisciplinary field that integrates sociological theory and design practice to critically explore how objects, systems, and futures shape and are shaped by social life. It provides both a theoretical lens and a methodological approach, through, of, and with design practices, to investigate sociomaterial relationships, promote social change, challenge normative assumptions, and support collaborative, future‐oriented research. Lupton (2018) argues that, depending on its application, design sociology can serve as a method for social critique and the identification of social inequalities. In this regard, design sociology aligns with IE in its shared focus on uncovering social inequalities and engaging in critical analysis of societal structures. However, to the best of the author's knowledge, there are no reported IE studies grounded in design sociology in the academic literature. The aim of this article is, therefore, to present and discuss design sociology as a framework for mapping and visualizing invisible work in IE studies. The article thus introduces a novel approach to conducting IE studies, proposing an innovative method for mapping, analyzing and presenting findings derived from IE research. In doing so, the article responds to previous research, which has highlighted that although mapping is frequently used in IE, there is a need for developing additional approaches to use mapping both as an analytical tool and as a means of presenting research findings derived from IE research (Hawkins 2024; Murray 2020). The rationale for using design sociology in IE studies is that design sociology offers creative and materially grounded methods for visualizing complex institutional processes and power relations, which is highly relevant to IE. As suggested in this article, this approach is valuable for making invisible work visible, as it facilitates both analytical depth and communicative clarity through tangible and critically reflective design practices.

The article is structured as follows: First, examples illustrating the practice of mapping in IE studies are presented. Next, the concept of invisible work is elaborated upon. This is followed by an introduction to design sociology. Building on a previous research project conducted in the elderly care sector, the article illustrates how design sociology can serve as a novel approach for mapping invisible work. Finally, the opportunities and challenges of applying design sociology in IE studies are discussed.

### 1.1 Mapping as Analytical Tool and Communication Medium

As previously mentioned, a key component of the methodology in IE involves mapping how people's experiences connect to institutions, policies, texts, and ruling relations. Smith (1999, 49) defines ruling relations as “that internally coordinated complex of administrative, managerial, professional, and discursive organization that regulates, organizes, governs, and otherwise controls our societies. It is not yet monolithic, but it is pervasive and pervasively interconnected.” Individuals both encounter and contribute to the reproduction of these ruling relations, which in turn structure and coordinate the organization of work. Building on this understanding of ruling relations, Smith (2005) further explores how these structures are identified and analyzed through the concept of the “problematic,” which guides the fieldwork in institutional ethnography. The initial problematic arises from a problem identified by informants; one that they are unable to solve, and cannot be fully explained by personal experience alone. Consequently, the researcher must consider the institutional context to gain a complete understanding (Rankin 2017).

Mapping ruling relations is an integral part of the ongoing inquiry process in IE studies and synthesizes the findings. Developing a literal map of ruling relations entails creating a graphical representation, sometimes described as constructing an artifact that elucidates how individuals are socially interconnected and how texts mediate these relationships (Smith 1987, 2005; Wright 2022).

DeVault (2014) suggests that mapping can be understood loosely and in a broad sense, encompassing the processes of noticing, analyzing, and narrating what people are doing. Some institutional ethnographers, however, employ more structured mapping techniques to present the findings of their research. With reference to Turner (2006), DeVault (2014) explains how mapping, for instance, can be represented through diagrams that illustrate complex activities. Turner's (2006) mapping procedure is detailed and extensive, visualized through circles and rectangles (Smith and Griffith 2022).

Karlsson et al. (2024), who illustrate the potential of mapping in their study of Patient and Public Involvement and Engagement (PPIE) in health research, show how visualizations serve as both an analytical tool and a communication medium. As an analytical tool, the mapping uncovers connections between institutional texts, ruling relations, and researchers’ practices, making institutional dynamics visible. As a communication medium, the visualizations and presentations of the mapping create “a window into informants' experiences and the social organization of researchers' work” Karlsson et al. 2024, 5, see also Smith and Griffith 2022.

Hawkins (2024) provides a further example by examining the challenges associated with implementing effective school food policies, focusing on the interaction between local practices and broader systemic forces. Utilizing IE and systems thinking, the study demonstrates how institutional and societal structures influence the work practices of school food staff. To address the limitations of traditional narrative approaches in IE, Hawkins integrates visual sketches and diagrams, offering a non‐linear representation of interdependencies, feedback loops, and power dynamics. This methodological approach, Hawkins (2024) argues, reveals underlying power structures and enhances the accessibility and applicability of research findings.

Ostridge (2025) investigates how ruling relations are enacted through the genealogy of a stand‐alone sexual violence policy at a Canadian university. Using IE, the study demonstrates the social organization of the policy‐making process, including its neoliberal framing and how it aligns institutional practices with legal and managerial imperatives. In the study, a conceptual map is utilized. This visual representation includes circles and boxes that signify key institutional texts alongside institutional actors, as well as processes that connect these elements. Arrows are used to link the various components, illustrating the relationships and workflows that reveal how texts and actors interact within the policy‐making process. The map also distinguishes between two spheres, labeled as the “Public/External Sphere” and the “Private/Internal Sphere,” to differentiate the broader public context from internal institutional mechanisms. Additionally, texts and actors are organized into distinct visual groupings, reflecting their roles and positions within the overarching process of sexual violence policy creation and enforcement (Ostridge 2025).

In summary, a common feature of many IE studies is the use of visual mapping, such as diagrams, conceptual maps, sketches or flowcharts, to depict the complex relationships between institutional texts, actors, and processes identified in the research. These visual representations frequently include elements such as circles, boxes, and arrows et cetera.

### 1.2 Invisible Work at Work

In IE, work is broadly understood as any paid or unpaid activity that individuals engage in as part of their daily lives, which is coordinated through texts, policies, relations, and institutional structures. This definition encompasses both visible tasks and the invisible or unacknowledged labor often overlooked in research (Smith 1987, 2005; DeVault 2006, 2014).

Increasing attention has been directed toward the study of invisible work in the labor market (Lavee and Kaplan 2022). Research shows that women, regardless of their professional achievements or hierarchical positions, are disproportionately expected to perform unpaid tasks across various sectors, as well as unpaid domestic labor. The growth of the service sector within a neoliberal framework has increasingly compelled women in caring professions to perform unpaid tasks as part of their paid employment. Care work has traditionally been regarded as an inherent responsibility of women, assumed to come naturally due to their gender, and therefore perceived as requiring minimal skill (Armstrong and Szebehely 2023; Baines et al. 2017). In the literature, invisible work in the workplace is sometimes described similarly to “office housework.” Office housework refers to tasks such as arranging coffee and snacks, providing emotional support to colleagues, managing interpersonal relationships, taking meeting notes, scheduling meetings, and so on. The invisible practices and work, that is, tasks that go beyond what the worker's position formally requires, can serve as core functions that are absolutely essential for the organization to operate. While beneficial to the organization, these tasks are often undervalued and do not directly contribute to career advancement. These activities can be considered informal practices and services that are unpaid, unregulated, and often invisible in official job descriptions or formal definitions of work (Lavee and Kaplan 2022; Jang et al. 2021).

The project discussed in this article serves as an example of how IE can draw on design sociology. It focuses on capturing the invisible work performed by caregivers in elderly care settings, that is, invisible work at work, as opposed to invisible work in the context of unpaid domestic labor. Before presenting the project and its context in greater detail, an overview of design sociology is provided.

### 1.3 Design Sociology

Design sociology is an interdisciplinary field that integrates sociological theories with design methods to address complex societal challenges. Its approach, as outlined by Lupton (2018), encompasses three main orientations: sociology of design, sociology through design, and sociology with design. Each orientation highlights different ways of intertwining design and sociology to understand and influence social structures and practices​. The sociology of design examines how design is shaped by sociocultural and political contexts, focusing on the practices, artifacts, and spaces within which designers operate. In contrast, sociology through design employs design as a methodological tool in sociological research, generating insights through the creation of design artifacts. Sociology with design involves collaborative efforts between sociologists and designers to explore societal phenomena​.

According to Lupton (2018), design sociology has the potential to address applied and future‐oriented research. By leveraging design‐based methods, sociologists can foster creativity, engage diverse stakeholders, and generate innovative ideas. For instance, Lupton's work on digital health employed participatory design workshops to map technological use and envision future innovations. Similarly, projects on personal data surveillance and education utilized speculative and participatory design to uncover social dynamics and imagine alternative futures​ (Lupton 2018).

Design sociology may draw on methodologies such as reflective design, speculative design, critical and norm‐critical design. These approaches challenge dominant norms, provoke critical questions, and explore speculative scenarios. For example, critical design raises awareness of social inequalities and explores future possibilities, while norm‐critical design addresses societal biases related to gender, age, and class. The aim of these design approaches is not to create a better product or service. Rather, the objective is to develop design concepts that challenge assumptions, provoke critical reflection, and foster discussion (Lupton 2018; see also Dunne and Raby 2013; Malpass 2013; Sengers et al. 2005).

In conclusion, design sociology offers a framework for integrating sociological and design perspectives to address contemporary issues. Its interdisciplinary nature holds potential for fostering collaboration, advancing sociological research, and promoting innovation in addressing social challenges.

### 1.4 Mapping and Visualizing Invisible Work at Work in the Elderly Care Sector

In a previous project, design sociology and IE were employed as methodological and theoretical frameworks. The project focused on uncovering the often‐overlooked and invisible labor within the female‐dominated elderly care sector. Following the principles outlined in IE (Smith 1987, 2005), our inquiry began with an exploration of caregivers' lived experiences in their everyday work context (Isaksson 2025). Through interviews and observations, we documented how their labor was characterized by exceptionally demanding workloads and poor working conditions. Moreover, their work was shaped by policies that, while well‐intentioned, ultimately proved counterproductive. These policies not only generated additional invisible labor but also exacerbated the already substantial workload faced by caregivers (Isaksson 2025).

While the initial empirical data collection was carried out using conventional qualitative observations and interviews, the subsequent stages of the mapping process were guided by design principles. In the project, I, as a sociologist, collaborated with researchers from backgrounds in design and political science. During the analysis of the interviews, simple sketches were created to visually represent selected examples of caregivers’ invisible work, actions, and experiences, conveying their insights through visual imagery rather than text. Drawing on IE, we then explored how these individual actions were coordinated within broader institutional processes. At a certain stage in the mapping process, it became evident that the caregivers’ experiences were consistently associated with some form of “bending the system.” In this context, “bending the system” refers to the caregivers’ repeated need to develop unofficial strategies to navigate around established structures, policies, and norms. They were required to maneuver within bureaucratic constraints while simultaneously responding to unrealistic work expectations. When “bending the system,” caregivers engaged in invisible work; tasks that were time‐consuming “workarounds,” necessary yet not officially recognized.

**Figure 1:**
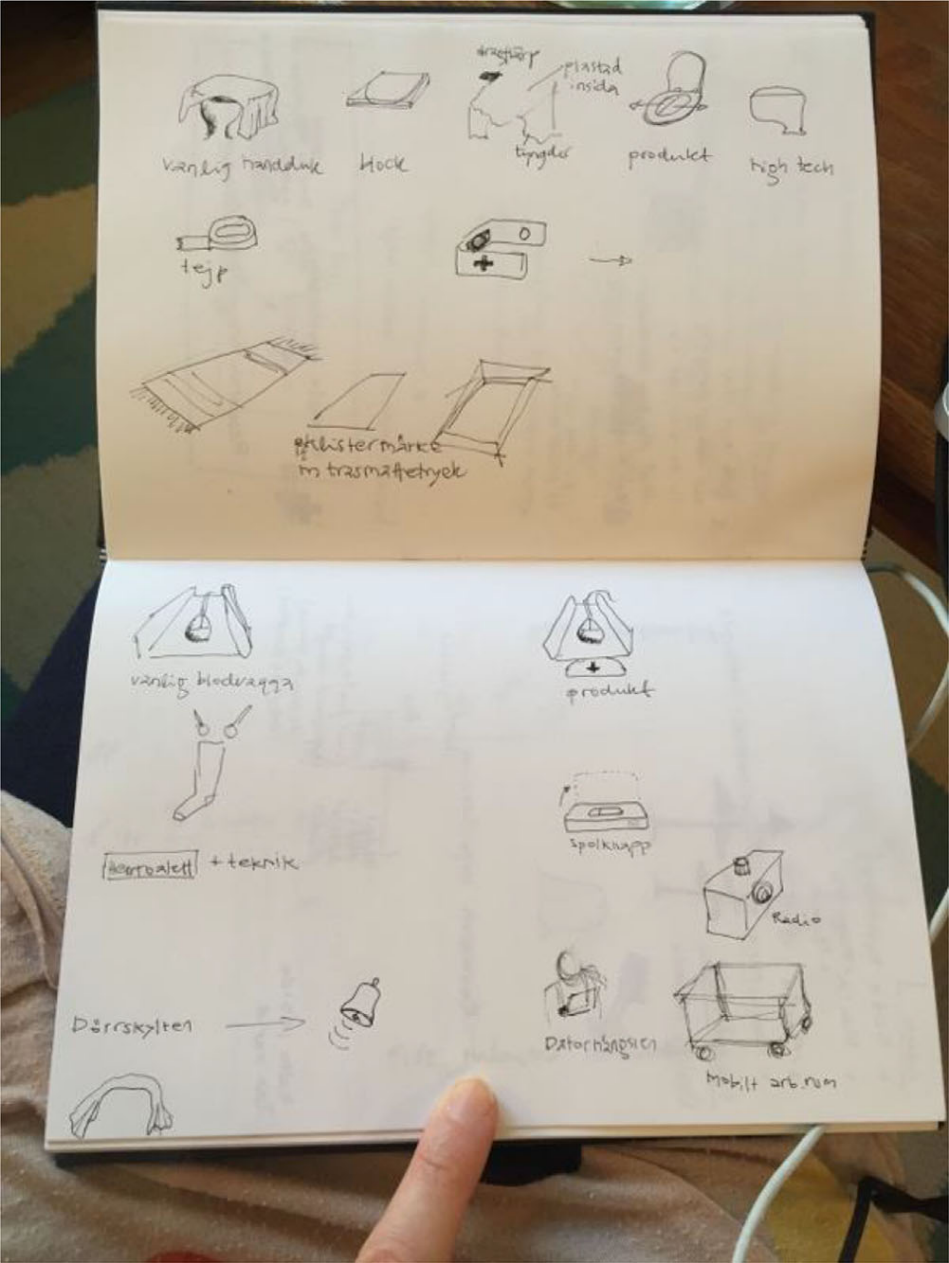
Early design sketches illustrating the iterative process of refining design concepts during the mapping phase, highlighting how these concepts evolved to address key institutional and caregiving challenges. The figure shows several examples of invisible work performed by the staff. In addition to early sketches of what became the elevator stopper, one example illustrates how staff would place rugs on the floor as “bridges” to help elderly individuals feel safe walking over sections of the floor with colour differences. This highlights a broader institutional and structural issue: that elderly care home buildings are not adapted to the needs of the elderly. Another example shows how staff would place a towel on the toilet seat and quickly remove it just before an elderly person sat down, as without the towel, individuals with dementia might think they will fall into a hole, as the water in the toilet bowl resembles one. [Colour figure can be viewed at wileyonlinelibrary.com]

In the next phase of the mapping process, inspired by critical, norm‐critical, and reflective design (Dunne and Raby 2013; Malpass 2013; Sengers et al. 2005), the visual sketches (see Figure 1) were not considered an endpoint. The caregivers provided us with several experiences and examples, and we chose to transform a selection of them into tangible objects. These objects were designed not only to represent experiences but also to illustrate their connection to the institutional processes and policies that regulate them, serving as a material representation of the mapping. Building on this idea of material representation, it can be argued that design sociology introduces a novel approach to mapping. The difference between “traditional” mapping in IE and mapping using design sociology is that, in a design sociology‐inspired study, visual elements are integrated into the mapping process from the outset. In traditional IE mapping, local experiences are typically connected to translocal/ruling relations through arrows, boxes, and diagrams. However, with the involvement of designers, mapping in design sociology links everyday experiences to the ruling relations that coordinate them by also creating and sketching potential and tangible design artifacts. These artifacts can be iterated through multiple stages as part of the research process, aiding in the understanding of how local experiences are connected to broader, translocal structures. In this project, the sketches were created by the researchers in an iterative process, during which the researchers communicated with the participants, that is, the staff, as the concepts developed.

The final objects were intended to foster discussion and reflection. Two examples illustrating the design concepts developed during the project are presented below, while additional examples can be found in Andersson et al. (2019).

### 1.5 The Elevator Stopper

The Elevator Stopper is a design concept inspired by caregivers' accounts in elder care, highlighting the challenge of preventing dementia residents from wandering, especially during understaffed night shifts. Locking people in is prohibited, so one unofficial practice involved disabling the elevator by taping its sensors while it remained in the basement, reducing the exits staff needed to monitor. Although common, such practices are not documented in official guidelines or protocols.

The elevator stopper was designed as a small, portable device that attaches to elevator sensors to prevent the doors from closing, making the elevator inoperable. By turning the informal workaround (taping sensors) into a tangible product, the design aimed to highlight caregivers' structural dilemmas. It raised critical questions about institutional responsibility: If such measures are necessary, should not there be a formal solution? The artifact also sparked discussions on where accountability for these systemic challenges should lie, whether at the administrative, institutional, or policy level (Andersson et al. 2019).

**Figure 2:**
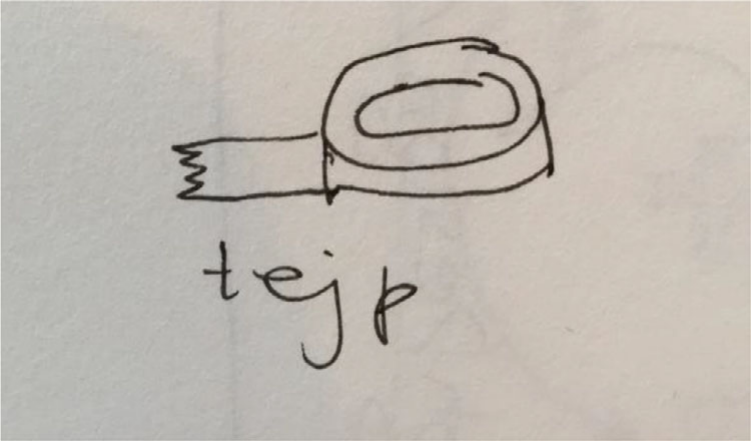
Early sketch of a simple tape solution used to cover the elevator's sensors while it remained in the basement, thereby reducing the number of exits staff needed to monitor. This mundane technical fix forms the basis of an invisible and unrecognized work task, as such practices are not documented in official guidelines or protocols. [Colour figure can be viewed at wileyonlinelibrary.com]

**Figure 3:**
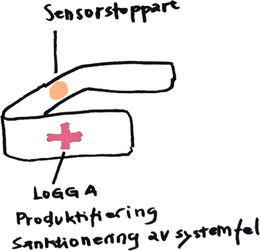
Iterative sketches of the elevator stopper concept, transforming the informal workaround of taping sensors into a tangible product that makes caregivers’ structural dilemmas visible and implicitly questions institutional responsibility for actions that are necessary but remain unrecognized. [Colour figure can be viewed at wileyonlinelibrary.com]

**Figure 4:**
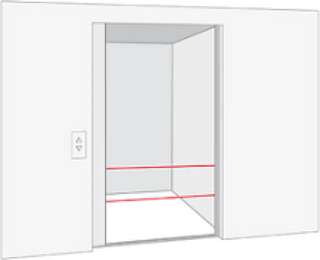
Prototype of the elevator stopper mounted on the elevator sensor. [Colour figure can be viewed at wileyonlinelibrary.com]

**Figure 5:**
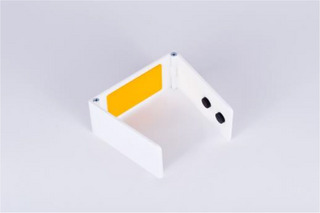
Close‐up of the final elevator stopper concept. Photo: David Molander. [Colour figure can be viewed at wileyonlinelibrary.com]

**Figure 6:**
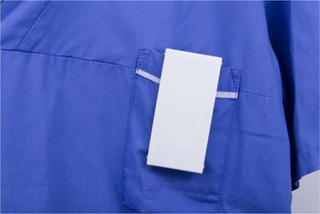
The elevator stopper in context, illustrating how it can be easily carried in a sweater pocket as an integrated part of everyday work and a materialization of otherwise invisible tasks. Photo: David Molander. [Colour figure can be viewed at wileyonlinelibrary.com]

By transforming this unspoken practice into a product, the elevator stopper made the issue visible and encouraged reflection on power dynamics and structural deficiencies in elder care. It highlighted how systemic constraints force caregivers into difficult decisions, shifting responsibility from individuals to broader institutional and political accountability. For instance, who would be willing to put their logo on such a product? In doing so, it connected caregivers' experiences with translocal and institutional processes (Andersson et al. 2019) (Figures 2, 3, 4, 5, 6).

### 1.6 The Client Generator

The client generator concept arose from challenges faced by home care staff using a digital route optimization system designed for the transportation sector. The system, developed for vehicles like cargo trucks, failed to account for conditions affecting staff traveling by bike or on foot, leading to discrepancies flagged as “deviations.” This highlighted a broader issue where digital tools regulate labor without considering human and environmental factors. In response, staff developed “workarounds,” such as creating fictitious clients in the system to adjust schedules to real conditions. To make this invisible workaround visible, the client generator was conceived as a physical device that prints a randomly generated fictitious client card, which staff could use to create more accurate schedules. This artifact exposes how caregivers compensate for poorly designed technologies, challenging the assumed neutrality of digital systems and highlighting their impact on labor relations and institutional accountability (Andersson et al. 2019).

**Figure 7:**
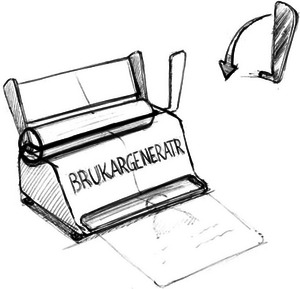
Early sketches of the client generator concept.

**Figure 8:**
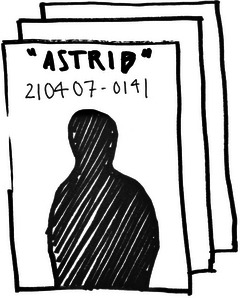
Early sketch of how a randomly generated fictitious client card could look.

**Figure 9:**
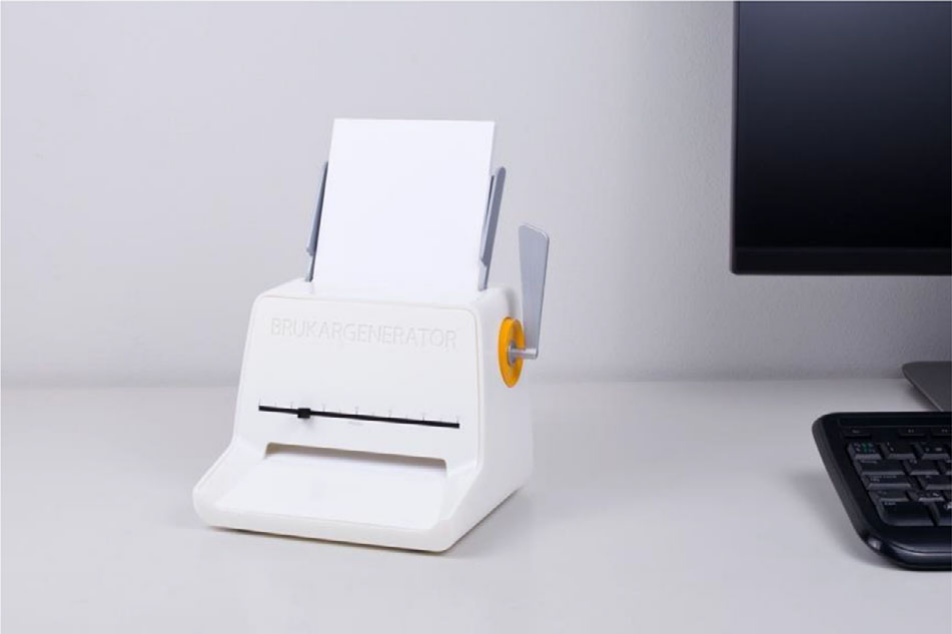
Final client generator prototype. Photo: David Molander. [Colour figure can be viewed at wileyonlinelibrary.com]

**Figure 10:**
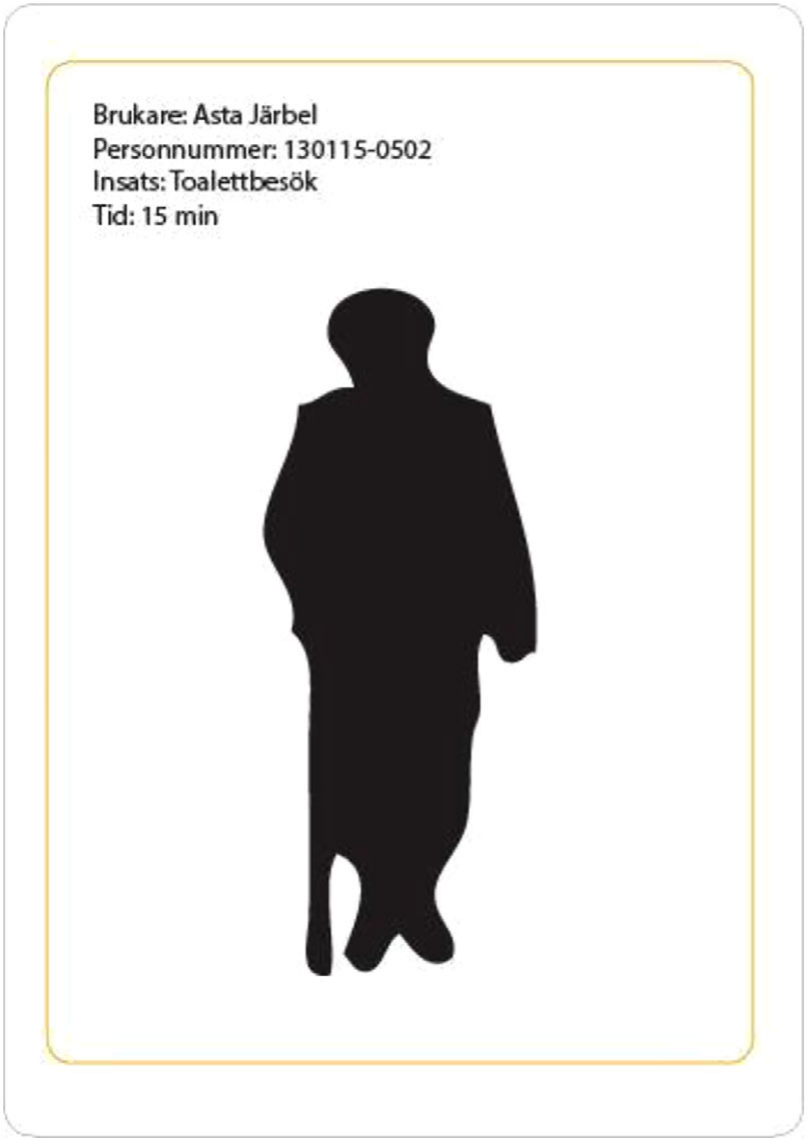
Final example of printed client cards generated by the device. [Colour figure can be viewed at wileyonlinelibrary.com]

Furthermore, the client generator visualizes the structural shortcomings of digital governance in elder care, prompting critical questions about system design, its beneficiaries, and the positioning of frontline workers within these structures. Instead of disrupting the system, the workaround reinforces its operation, reflecting broader resource constraints and limited worker agency in technological decisions (Figures 7, 8, 9, 10).

## 2 Discussion—The Potential and Challenges of Applying Design Sociology in IE Studies

Informed by a previous research project conducted in the elderly care sector, this article has demonstrated how design sociology can function as a methodological tool for mapping and visualizing invisible work within IE studies. By integrating iterative design techniques and transforming experiences, along with their connections to institutional and translocal processes, into tangible design concepts, this approach has the potential to expand the methodological scope of IE research. It offers new strategies for both data analysis and the presentation of findings. Although the importance of mapping in IE is widely acknowledged, previous research highlights opportunities for developing additional mapping approaches (Hawkins 2024; Murray 2020. This article has demonstrated how design sociology can address this need by offering a “designerly” (c.f. Lupton 2018) approach to mapping, both as an analytical tool and as a medium for research communication. In doing so, it contributes to the ongoing development of IE methodologies by proposing new techniques for critically examining, mapping and visualizing how experiences are shaped by institutional processes. As demonstrated in this case, it also offers methods for materializing invisible work. Sharing methodological insights in IE, as exemplified in this article, is not about advocating for methodological dogmatism. Rather, it seeks to advance a pluralistic repertoire of research practices (Smith 2005; Rankin 2017).

However, like any interdisciplinary aspiration, design sociology may pose complexities for sociologists, particularly in its practical application. Engaging with design methodologies requires a shift in perspective, one that may not come naturally to those trained in traditional sociological approaches. As Lupton (2018, 8) notes:

Sociologists have not been trained to think in “designerly” ways. Most need help to conceptualise how to go about incorporating design perspectives into their research: and this means collaboration outside sociology. Thus far, of the small number of sociologists who have experimented with design research, most have training in design as well as sociology or have used designers as consultants, or worked on multidisciplinary research teams including designers or HCI researchers. Sociologists who want to incorporate design research approaches but have little hands‐on experience of them may need to consider making connections with academics working in design or design anthropology or commission design consultants who have experience in the types of design research outlined here to work with them in planning and executing their projects.

Collaborating with researchers with a background in design has been crucial for me in applying design research and design thinking into various research projects. As with any interdisciplinary collaboration, challenges arise when different disciplines converge. However, I believe that working with designers has enabled me to uphold one of the foundational principles of IE, ensuring that research findings remain grounded in data rather than being abstracted into theory (cf. Rankin 2017). Rankin (2017, 2, original emphasis) reminds us that:

(…) the research goal is to *empirically link, describe, and explicate tensions embedded in people's practices* not to theorize them. While this core tenet may seem dogmatic, it is what makes an IE an IE. Work that fails to adhere to this foundational precept fails as an IE.

I argue that the design‐oriented mapping process, converging in visual and tangible design concepts, prevents research from getting lost in abstract theory. The iterative and visual nature of design helps maintain contextual sensitivity, ensuring that insights remain closely tied to their empirical foundations. Furthermore, design artifacts serve as material representations of ideas, reducing the risk of drifting into abstract theorization disconnected from the original data (Isaksson 2025). As such, the close ties between research findings and the experiences of participants are preserved (cf. Smith 2005; Rankin 2017).

## 3 Concluding Reflections

The aim of this article has been to present and discuss design sociology as a framework for mapping and visualizing invisible work in IE studies. By applying design sociology in an IE‐oriented study in the elderly care, this article has demonstrated how design methodologies can expand the methodological repertoire of IE, offering novel and innovative ways to map, visualize, and communicate how experiences are shaped by institutional and structural processes. At the same time, IE's strong commitment to grounding research in people's experiences provides a valuable sociological foundation for design sociology, strengthening its analytical depth and critical engagement with institutional structures. This reciprocal exchange highlights the potential for further interdisciplinary development, where both fields can borrow from and enrich each other's methodological and theoretical frameworks.

## Author Contribution

Anna Isaksson is the sole author and was responsible for the design, analysis, and all stages of manuscript preparation, including drafting and revisions in response to reviewers' comments.

## Conflicts of Interest

The author declares no conflict of interest.

## Funding

The author received financial support for the research and writing of this article from the REBEL research program at Halmstad University.
